# From Herd Health to Public Health: Digital Tools for Combating Antibiotic Resistance in Dairy Farms

**DOI:** 10.3390/antibiotics13070634

**Published:** 2024-07-09

**Authors:** Andra-Sabina Neculai-Valeanu, Adina-Mirela Ariton, Ciprian Radu, Ioana Porosnicu, Catalina Sanduleanu, Gabriela Amariții

**Affiliations:** 1 Research and Development Station for Cattle Breeding Dancu, 707252 Iasi, Romania; sabina.valeanu@scdb-dancu.ro (A.-S.N.-V.); mirela.ariton@scdb-dancu.ro (A.-M.A.);; 2 The Academy of Romanian Scientists, Str. Ilfov No. 3, Sector 5, 050045 Bucharest, Romania; 3 Faculty of Veterinary Medicine, Iasi University of Life Science, 700490 Iasi, Romania; 4 Faculty of Food and Animal Resources, Iasi University of Life Science, 700490 Iasi, Romania

**Keywords:** antimicrobial resistance, dairy farming, digital health monitoring, precision livestock farming, sustainability

## Abstract

The emergence of antimicrobial resistance (AMR) is a significant threat to global food security, human health, and the future of livestock production. Higher rates of antimicrobial use in dairy farming and the sheer lack of new antimicrobials available for use focused attention on the question of how the dairy production sector contributed to the development of AMR and paved the path toward taking action to curtail it on the targeted type of farms. This paper aims to provide an introduction to a phenomenon that has gained considerable attention in the recent past due to its ever-increasing impact, the use of antimicrobial drugs, the emergence of antimicrobial resistance (AMR) on dairy farms, and seeks to discuss the possibilities of approaches such as digital health monitoring and precision livestock farming. Using sensors, data, knowledge, automation, etc., digital health monitoring, as well as Precision Livestock Farming (PLF), is expected to enhance health control and minimize disease and antimicrobial usage. The work presents a literature review on the current status and trends of AMR in dairy farms, an understanding of the concept of digital health monitoring and PLF, and the presentation and usefulness of digital health monitoring and PLF in preventing AMR. The study also analyses the strengths and weaknesses of adopting and incorporating digital technologies and artificial intelligence for dairy farming and presents areas for further study and level of use.

## 1. Introduction

One of the most pressing issues in the present world is antimicrobial resistance (AMR), which poses a threat to human, animal, and livestock lives. Some of the factors that have been regarded to have contributed to the development of antibiotic-resistant bacteria include the misuse of drugs in the food industry, especially dairy farming, and improper biosecurity on the farm [1,2]. Antibiotics are administered to animals for various purposes, not only for the therapy and control of certain diseases, but also for preventing such diseases as well. Prior research documented antibiotic-resistant strains such as methicillin-resistant Staphylococcus aureus (MRSA) in cattle [3,4,5]. Additionally, there are differences between organic and conventional dairies in terms of management practices regarding the use of antibiotics [6]. Tackling AMR in dairy farms is an important factor that cannot be overlooked and requires policies and measures. Different animal populations, especially livestock, are known sources of AMR that can be easily transferred to the human population. Therefore, there is a great need to develop policies and mechanisms for the implementation of antimicrobials in animals and to frequently assess the levels of resistance to them [3,7]. 

Unfortunately, prior studies have shown that dairy producers may lack awareness of the connection between antibiotic use and the emergence of antibiotic resistance [8,9,10,11,12,13]. Thus, long-term measures for handling antibiotic resistance should be taken and applied in the dairy industry. Smart health control is a more contemporary and efficient concept that uses technological tools like sensors, data and information analysis, and automation in the monitoring of livestock production systems [14,15,16]. This new concept is intended to improve animal well-being, output, and health through the reduced use of holds, such as antibiotics. The above has been made possible by wearable sensors that track animal health status by monitoring small signs such as body temperature, rumination, and activity [3,15,16,17,18]. These sensors collect real-time data on individual animal health, allowing farmers to detect possible health issues early on. Early detection of diseases or infections enables targeted interventions, such as isolation of sick animals, targeted treatment, and improved biosecurity measures, which can help prevent the spread of antibiotic-resistant bacteria within the herd. This proactive strategy lowers the demand for antibiotics and the possibility of antimicrobial resistance emergence [3,17,18]. 

Furthermore, this technology may facilitate the implementation of precision medicine approaches in dairy farming. By integrating data from individual animals, such as genetic information, health records, and environmental conditions, farmers can tailor treatment strategies to specific animals or groups, minimizing the use of broad-spectrum antibiotics and reducing selection pressure for antibiotic resistance. In addition to smart health, other technological advancements can help tackle AMR in dairy farms. Internet of Things (IoT)-based intelligent solutions, combined with blockchain technology, can enhance livestock management by providing real-time information on animal health and facilitating traceability [19]. These technologies, which are part of the precision livestock farming (PLF) concept, enable farmers to monitor health symptoms and manage livestock more effectively, thereby reducing reliance on antimicrobials.

These are important characteristics that help maintain high levels of tidiness and cleanliness to reduce disease prevalence. Measures for biosecurity have recently become important for controlling the spread of antibiotic-resistant bacteria in dairy farms. Stringent sanitary measures, proper disposal methods, and limited human contact with the animals explain why bacterial transmission may be heavily reduced [20,21,22]. The adoption of PLF in similar cases may enhance the effectiveness of such accurate biosecurity measures. Other measures may include entry control systems such as gates for vehicles and persons which help to reduce the incidence of entry by unauthorized persons and livestock that may spread antibiotic-resistant germs. In addition, the application of PLF systems may enhance the origin tracking of animals and food products with the potential to prevent and control disease spreading [23].

Therefore, to sustain dairy farming in the future and to safeguard the health of consumers, it is crucial to put in place certain measures that can help prevent the spread of AMR on farms. Thus, these solutions should consider reducing the use of antimicrobials while preserving the well-being and productivity of animals. This paper seeks to examine the possibilities of using smart health and precision livestock farming (PLF) as viable approaches to achieving these objectives.

## 2. Current State of AMR in Dairy Farming

Milk production is generally recognized for its high intensity, which leads to the provision of the right conditions for the development of antibiotic-resistant bacteria. A further factor that creates selection pressure that results in the appearance of resistant strains is the application of antimicrobials in dairy cattle to prevent and cure diseases. In addition, previous use of antimicrobial drugs for growth promotion led to the development of antibiotic resistance. Antimicrobials have traditionally been used as growth enhancers in cattle production, and this practice has been widespread in several nations. For example, in the United States, a significant proportion of feedlots have previously used antimicrobials to promote growth or prevent disease [24,25]. Similarly, in countries such as Nigeria, a significant proportion of intensive and semi-intensive farmers still use antimicrobials as growth promoters in beef production [26]. Studies have highlighted the use of antimicrobials in Ethiopia for therapeutic and prophylactic purposes in food animals, including cattle [27]. In regions like South America, a considerable portion of beef cattle are finished confined feeding operations and have historically been administered antimicrobial agents in feeds as growth promoters [28,29].

In recent years, there has been a worldwide change in regulations concerning the use of antimicrobials to enhance the development of cattle. In 2006, the European Union prohibited the use of antimicrobials to promote growth in animal production systems. The United States followed likewise in 2017. Despite being poorly received among animal farming groups, the implementation of these new restrictions has resulted in a significant decrease in the use of antimicrobials in livestock feeds across several countries [30,31]. This section highlights common dairy cow ailments that require antibiotic treatment, such as mastitis, lameness, and respiratory and digestive system disorders. The third section examines and describes dairy farmers’ views and actions regarding antibiotic use and its impact on population health.

### 2.1. Main Causes for Antibiotic Use in Dairy Farms

Possible factors related to antibiotic intake in dairy farms include farm size, the area in which the farm is located, and the management system of the farm. For instance, large-scale dairy farms will apply more antibiotics because they have many animals and the risk of disease is high [32,33,34,35,36,37,38,39]. Geographic regions can also play a role, as antibiotic usage patterns can vary between regions. Furthermore, certain management interventions such as the application of dry cow and clinical mastitis therapies could alter the type and frequency of antibiotic application. Mastitis is one of the most widespread diseases in dairy farms, affecting the udders of cows, and is an inflammatory disease that is mainly caused by bacteria, but viruses, fungi, and yeast may also be responsible for the disease [40]. The disease is one of the most important challenges in the dairy sector, resulting in financial losses owing to reduced milk production and higher treatment expenses [41]. This condition can manifest in various forms, including clinical, subclinical, and chronic mastitis [42]. Intra-mammary and/or systemic antibiotics are used to combat infection and limit the further diffusion of the bacteria in cows. The goals that can be achieved with antibiotics administration in mastitis treatment include improving the quality of life of animals, reducing disease incidence, and maintaining milk production.

Several pathogens are commonly associated with bovine mastitis, including *Staphylococcus aureus*, *Escherichia coli, Klebsiella pneumoniae*, *Streptococcus agalactiae*, and *Streptococcus uberis* [43]. *Staphylococcus aureus*, in particular, is a major agent of bovine mastitis and is known for its ability to acquire antimicrobial resistance and secrete virulence factors that exacerbate inflammation [44,45]. Methicillin-resistant staphylococci (MRS) are also increasingly isolated from bovine mastitis, posing challenges for treatment [46,47].

Different pathogens may require specific antibiotic treatments. For instance, *Staphylococcus aureus* infections in bovine mastitis have become less responsive to antimicrobial therapy because of widespread drug resistance [45]. The use of antibiotics such as kanamycin and cephalexin has been evaluated for their efficacy against *Streptococcus dysgalactiae*, another common mastitis pathogen [48]. For *Streptococcus uberis*, a pathogen that causes both clinical and sub-clinical mastitis in dairy cattle, Penicillin is considered a first-line antibiotic for treatment in some countries like Switzerland and the Czech Republic [49,50]. *S. dysgalactiae* is also often penicillin-sensitive, and effective therapy comprises penicillin or a third-generation cephalosporin, whereas fluoroquinolone and cephalosporins are the only antimicrobials that have shown therapeutic effects on mastitis caused by *Escherichia coli*. However, their use is indicated only in mastitis cases with severe clinical symptoms to avoid bacteremia and the unlimited proliferation of bacteria [51,52].

Additionally, *Klebsiella pneumoniae*-induced mastitis is a growing concern, with implications for both animal and public health through dairy products [53,54]. Understanding the pathogenicity and characteristics of mastitis-causing pathogens is critical for effective treatment. Studies have highlighted the importance of virulence factors, genetic variation, and antimicrobial resistance profiles in pathogens like *Staphylococcus aureus* and *Escherichia coli* associated with bovine mastitis [55,56]. Furthermore, the association between specific genotypes, such as rpoB sequence type, and the severity of mastitis caused by *Staphylococcus aureus* underscores the need for tailored treatment approaches [57].

Zhang et al. (2022) [45] highlighted that the high incidence of bovine mastitis is directly associated with the contamination of milk by antibiotics. The authors concluded that by implementing good farm practices to decrease the incidence of mastitis, there might be a decrease in the use of antibiotics in milk, thereby enhancing the safety of dairy products. The causes of mastitis should also be looked at considering the environment in which the disease occurs. Isolates of environmental streptococci present in bovine milk samples have been found to exhibit fluctuations in antimicrobial resistance profiles, implying the need for continued study and surveillance of the phenomenon for administrational interventions [58].

Thus, the management of bovine mastitis requires a multifaceted approach that considers pathogen-specific treatment strategies and environmental factors. The therapeutic approach for bovine mastitis should include consideration of the different types, including clinical, subclinical, and chronic forms. Subclinical mastitis is challenging to detect and often requires a combination of approaches. Research by Ji et al. (2022) [59] highlighted the difficulty of identifying subclinical mastitis because of the lack of obvious symptoms. Effective treatment may involve the use of rapid diagnostic tests to differentiate between bacterial types, as suggested by Speksnijder et al. (2024) [60], thereby aiding targeted antimicrobial therapy. Comparative studies have shown that both critically and non-critically important antimicrobials have comparable efficacy for treating non-severe clinical mastitis in dairy cattle [61].

For clinical mastitis, which presents with visible symptoms, antibiotics are commonly used along with supportive therapies, such as fluids and anti-inflammatory drugs, which are essential components of the treatment regimen. Chronic mastitis, which is characterized by persistent or recurrent infections, may require specialized treatment. Studies like that by Hoernig et al. (2016) [62] explored innovative therapies like lysostaphin-fusion proteins for chronic, subclinical *Staphylococcus aureus* mastitis. Furthermore, research by Lange-Consiglio et al. (2014) [63] suggests that chronic mastitis cases may require unconventional therapies like platelet concentrate. In cases of chronic mastitis that do not respond to conventional treatments, alternative approaches may be considered. Petitclerc et al. (2007) [64] evaluated novel treatments, such as lactoferrin-penicillin combinations for beta-lactam-resistant *Staphylococcus aureus* mastitis, whereas Zaatout et al. (2020) [65], Fidelis et al. (2024) [66], discussed the challenges posed by biofilm formation in chronic mastitis, indicating the need for tailored treatment strategies.

**Figure 1:**
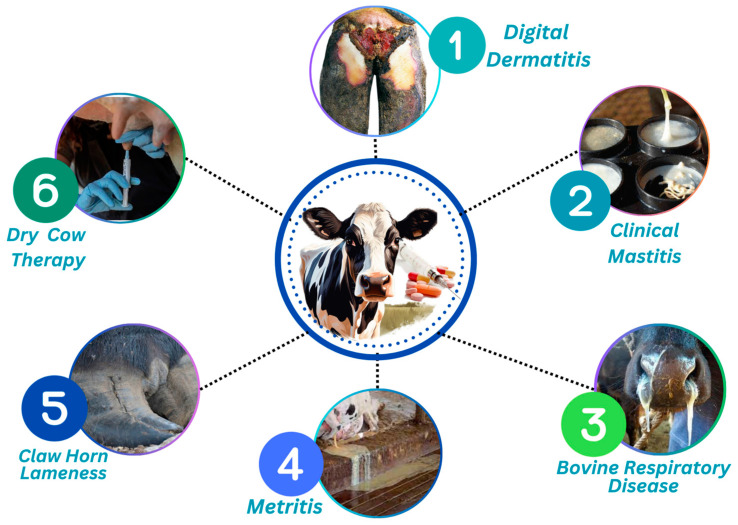
Main Causes of Antibiotic Use in Dairy Farms.

In addition to mastitis, antibiotics are administered to dairy farms to treat other bacterial diseases, including enteritis, foot rot, pneumonia, and metritis. These conditions may have an impact on the health and production of dairy cows, and antibiotics are used to prevent or cure them (Figure 1).

However, dairy cows are not the only antibiotic-resistant strains on farms. A study conducted by Salerno et al. (2022) [67] has pointed out that calves are the primary source of ARGs on dairy farms, and a correlation was noted between the usage of certain antibiotics, including penicillin, and certain ARGs. 

According to Mulchandani et al. (2023) [68], in 2020, China, Brazil, India, the USA, and Australia were the leading consumers of antimicrobials for veterinary use. Collectively, these nations accounted for 58% of global antimicrobial use. Furthermore, it is projected that they will continue to be the top five countries in terms of antimicrobial use (AMU) in 2030. In the EU, on the other hand, in 2021, for the first time, the population-weighted mean antimicrobial use (AMU) in food-producing animals was lower than that of people [69]. This decrease in AMU in food-producing animals could be explained by the legislation and stewardship initiatives developed by the EU and implemented accordingly in the member states.

### 2.2. Classes of Antibiotics Used in Cattle Production and Therapeutical Alternatives

The worldwide AMU (tonnes) for cattle, sheep, poultry, and swine in 2020 was anticipated to be 99,502 tonnes of the active ingredient, with the usage per biomass unit for cattle estimated at 59.6 mg/PCU. The use of antibiotics is necessary for the dairy herd to combat diseases such as enteritis, mastitis, or cattle tick fever and to protect the animals’ health [70]; nevertheless, the excessive and/or improper application of antibiotics in cattle farming has severe repercussions on the formation of antibiotic resistance, which underlines the significance of the proper utilization of antibiotics in the dairy industry [71,72,73] and the implementation of proper antimicrobial stewardship programs to prevent the further emergence of antibiotic resistance. Tetracyclines and penicillin are the most commonly used antibiotics in animals. In 2018, European nations administered 31.7 mg/PCU (Population Correction Unit) of tetracyclines and 29.7 mg/PCU of penicillin for veterinary medicine. These drugs were used at up to a 30-fold rate compared with other antibiotic groups [68]. In cattle, tetracyclines have been used in cattle for decades to treat a broad range of bacterial skin diseases, including multidrug-resistant pathogens like MRSA [74]. Chlortetracycline and oxytetracycline are extensively and effectively used to prevent and treat bovine pneumonia, calf scours, foot rot, metritis, and acute mastitis, as well as *Pasteurella multocida*-associated infections.

First-generation cephalosporins like cefazolin, and second-generation cephalosporins such as cefprozil, are widely used for treating mastitis in dairy cattle [75]. Other antibiotics approved for mastitis treatment include streptomycin, ampicillin, cloxacillin, and penicillin [76,77]. For respiratory disorders in cattle, antibiotics like tilmicosin, florfenicol, enrofloxacin, and ceftiofur are commonly employed for the treatment and prevention of bovine respiratory disease [78]. Beta-lactam antibiotics are predominantly used for various conditions in cattle, including mastitis, pneumonia, and metritis. Ceftiofur, a third-generation cephalosporin, has been specifically approved for treating mastitis infections in cattle [79]. However, it is also routinely used as an effective treatment for foot rot and bovine respiratory disease (BRD). 

Aside from tetracyclines and β-lactams, other commonly used types of antibacterial agents for treating bacterial skin disorders in cattle include macrolides, aminoglycosides, and fluoroquinolone. In addition, specialized compounds such as florfenicol are also employed. Novel antibiotic classes like rationally designed pyrimidine compounds and nanohybrid antibiotics, are also being explored for treating mastitis in cattle [80,81]. The selection of antimicrobial agents for dairy cattle is typically based on their effectiveness and common usage in the industry [82]. The evaluation of antimicrobial use in dairy cattle often involves indicators like antimicrobial treatment incidence, providing insights into the frequency of antimicrobial use on dairy farms [83].

The use of antibiotics is necessary in the dairy herd to combat diseases such as enteritis, mastitis, or cattle tick fever and to protect the animals’ health [70]; nevertheless, the excessive and/or improper application of antibiotics in cattle farming has severe repercussions on the formation of antibiotic resistance, which underlines the significance of the proper utilization of antibiotics in the dairy industry [71,72,73] and the implementation of proper antimicrobial stewardship programs to prevent the further emergence of antibiotic resistance.

Knowledge of the pharmacokinetic/pharmacodynamic (PK/PD) characteristics of antibacterial agents is also essential in the maximization of the effectiveness of drugs while avoiding instances of misuse through wrong dosing regimens. According to Nielsen et al. (2011) [84], data obtained from in vitro and animal models are useful for explaining the correlation between PK and PD of antibacterial agents. For example, Zhou and his team (2017) [85] found that the Index PK/PD is useful for comparing the effectiveness of tulathromycin against *Pasteurella multocida* as the ratio of the concentration of the drug within the bloodstream over 24 h to the minimum inhibitory concentration, thus AUC(0–24 h)/MIC. Regarding veterinary antimicrobial susceptibility testing, the determination of clinical breakpoints (CBPs) for antibacterial drugs is essential [86]. Thus, these CBPs assist in setting the concentration of an antibiotic distinguishing between a susceptible and a resistant pathogen, which is beneficial for treating and fighting AMR. Furthermore, PK/PD models are widely applied to describe the effectiveness of antibacterial agents in different infection models and optimize the dose and therapeutic outcomes [87]. A combination of PK and PD parameters is appropriate for assessing the antibacterial efficacy of cefquinome against pathogens such as Staphylococcus aureus, in mastitis cases [88]. Indexes including the time the drug concentration remains above the minimum inhibitory concentration (T > MIC) and the area under the curve to MIC ratio (AUC/MIC) are important indices for measuring antibacterial activity. Likewise, for tulathromycin, the 24-h AUC/MIC ratio in serum is a suitable PK/PD parameter that reflects antibacterial efficacy [88].

Therefore, in the veterinary field, identifying the PK/PD profiles of antibacterial agents is crucial for developing appropriate therapy regimens for diseases such as bovine mastitis caused by Staphylococcus aureus [89]. Researchers have pointed out that PK/PD modeling can be used to evaluate the antibacterial efficacy of different drugs against particular pathogens, such as isopropoxy benzene guanidine against *Clostridium perfringens* in broilers for example [90]. These models provide valuable insights into optimal dosing regimens and treatment outcomes for different animal species, including cattle. Furthermore, the pharmacokinetic considerations of antibiotics in critically ill animals can significantly impact their PK/PD profiles. Factors like altered protein levels and changes in the volume of distribution and clearance can influence the efficacy and dosing strategies of antibacterial agents. Therefore, tailoring antibiotic regimens based on a thorough understanding of the PK–PD relationships is crucial for ensuring optimal treatment outcomes in diverse clinical scenarios. The integration of pharmacokinetic and pharmacodynamic principles is essential for evaluating the efficacy of antibacterial agents used in cattle production. By leveraging PK/PD modeling and understanding critical parameters like AUC/MIC ratios, %T > MIC, and AUC24 h/MIC, veterinarians can optimize dosing regimens, enhance treatment outcomes, and mitigate the development of AMR in livestock settings [91]. 

Exploring non-antimicrobial interventions to mitigate AMR in cattle production systems could help decrease the occurrence of resistance. Over the past decade, there has been a growing interest in the use of alternative and complementary therapies in the management of livestock health and welfare, particularly in the dairy industry where consumers are increasingly demanding more natural and sustainable approaches to animal care. Research on alternative treatments with increased effectiveness and reduced costs may contribute to responsible antibiotic use in dairy farms. 

One potential alternative to antibiotics is the use of bacteriophage endolysins, which have a broad lytic spectrum targeting multiple genera and could be effective in treating bovine mastitis [92]. Bacteriophages are viruses that infect bacteria and have shown potential as alternatives to antibiotics for the prevention and treatment of diseases in cattle [93]. These phages can target specific bacterial pathogens, reducing the need for broad-spectrum antibiotics and minimizing the selection pressure for antibiotic resistance. Moreover, the use of bacteriophages isolated from dairy farms has shown promise in mitigating inflammation caused by mastitis pathogens, offering a novel treatment approach [94,95,96].

Probiotics such as *Sporidiobolus ruineniae* have demonstrated antimicrobial activity against pathogens like *Staphylococcus aureus* [97]. Incorporating probiotics such as lactic acid-utilizing yeasts into dairy farming practices to reduce ruminal lactic acid production can promote a healthy microbiome and implicitly reduce the reliance on antibiotics [98,99].

Another area that has garnered significant attention is the use of essential oils (EOs) for the treatment and prevention of diseases in dairy cattle. Essential oils are highly concentrated aromatic compounds extracted from various plant materials and have been shown to possess a wide range of pharmacological properties, including antibacterial, antiviral, and anti-inflammatory effects [100,101,102]. A study conducted by Caneschi et al. (2023) [103] identified factors influencing the efficacy of teat dips and explored the use of essential oils and natural compounds like geraniol as potential alternatives to antibiotics for managing mastitis without inducing resistance. Moreover, a pharmacoeconomic analysis of conventional antibiotic treatment versus the proposed Phyto-Bomat treatment based on essential oils in bovine mastitis therapy was conducted in 2022 by Kovačević et al. (2022) [104]. Their analysis compared the costs and effectiveness of the two treatments, providing insights into potential alternatives to conventional antibiotic therapy. Ethanol extracts from natural sources such as oak bark, heather herbs, moringa, and garlic also provide antimicrobial properties against pathogens found to cause bovine mastitis [105] and endometritis in dairy cattle [106,107].

Furthermore, the integration of nanotechnology in veterinary medicine offers a multifaceted approach to enhancing the health and well-being of dairy cattle. Nano-particles, materials with at least one dimension in the nanometer range (1–100 nanometers), exhibit unique physical and chemical properties compared to their bulk counterparts and offer promising solutions for addressing various health challenges in dairy cattle, including diseases like mastitis, endometritis, lameness, and respiratory infections [100,108]. Chitosan nanoparticles have antimicrobial and antibiofilm effects against bacterial isolates obtained from mastitis-affected cows [109], whereas stable silver nanoparticles and quercetin of plant origin expressed the antibacterial and anti-biofilm action against mastitis-associated microorganisms [110]. Innovative therapeutic approaches based on nanotechnology are developed for other conditions. Amin et al. (2023) [111] proposed a new therapy for postpartum endometritis caused by multi-drug-resistant bacteria based on green-generated zinc oxide nanoparticles (GZnO NPs). The GZnO NPs displayed significant effectiveness against multidrug-resistant bacteria in postpartum endometritis both in vitro and in vivo. Zinc oxide nanoparticles (NPs) effectively reduced the presence of *Escherichia coli* and *Staphylococcus aureus* in the uterus, leading to a high success rate in treating the infection. The unique properties of silver nanoparticles have also been explored, either alone or in conjunction with bacteriophages, as a potential strategy for combating multidrug-resistant bacteria in dairy cattle. In a study conducted by El-Sayed et al. (2023) [112], bacteriophages targeting *Listeria monocytogenes* from dairy cattle farms were isolated, and their antimicrobial effects were assessed in combination with silver nanoparticles. This approach demonstrates an innovative use of nanotechnology to enhance the efficacy of bacteriophages in controlling bacterial infections.

### 2.3. The Attitudes and Practices of Dairy Farmers Concerning Antibiotic Use

Several factors affect the perception and practices of dairy farmers regarding antibiotic use. A quantitative study by Jones et al. in 2015 [9] showed that the perception of veterinarians toward antibiotics determines the farmer’s decision. This study also identified that most farmers either reduced the usage of antibiotics in recent years or planned to reduce the usage in future years. This indicates that society is in the process of being sensitized to the importance of using antibiotics as recommended. In the other research conducted by Casseri et al. (2022) [71], the authors focused on the level of concern that dairy farmers have about antibiotic resistance and their understanding of the new tactics that can be used to regulate antibiotic use. Most participants felt that the concept of antibiotic resistance caused by the overuse of antibiotics is either questionable or completely false. Therefore, educational measures are needed to correct misconceptions and encourage correct antibiotic consumption.

Furthermore, quantitative research conducted in New York State examined the perceptions of dairy farmers about antibiotic resistance [8]. The findings showed that most dairy farmers did not have concerns about the misuse of antibiotics in cattle, which in one way or another affected the farmers themselves to develop antibiotic resistance. This lack of concern among some dairy producers is critical in pointing out the need to create awareness among the public on the effects of administering antibiotics to dairy cattle. This conclusion was supported further by a study conducted in the Indian states of Assam and Haryana [113]. The research design used in the study was a cross-sectional survey that incorporated both qualitative and quantitative data collection methods of interviewing farmers, farm assessment, and milk analysis. The results suggested that antibiotic use on farms was presumably not very high because only 10% of the farmers confessed that they had used antibiotics on their dairy cows. However, the aforementioned study identified that approximately 8% of the farms included in the study had milk samples that were positive for antibiotic residues, indicating that there is a need to assess the use of antibiotics and the residues on dairy farms to ensure the production of safe milk [113].

Intensive dairy production is linked to increased antibiotic use in management practice. One study in central Italy examined farms with beef and dairy cattle and determined that various practices led to increased antibiotic use. Therefore, enhancing the management practices of dairy farms could help decrease the use of antibiotics [114]. In addition, a longitudinal study on antibiotic use in dairy and beef cattle farms in Germany has provided information on the treatment patterns and frequency [115]. This study’s data were collected from 2011 to 2015 and helped illuminate the usage pattern of antibiotics among different categories of cattle. In the authors’ view, assessing antibiotic consumption on dairy farms is essential for detecting long-term trends and developments. 

Furthermore, it is also important to know the perception and attitude of dairy farmers regarding the use of antibiotics because this is a basis for devising measures that will promote the appropriate use of antibiotics. Georgakakos et al., 2021 [116] conducted a qualitative interview study that aimed to understand the perceptions of dairy farmers regarding antibiotic use and resistance. This study established that farmers were aware of the difference between the expectations of society and the government regarding the use of antibiotics and their actions. This shows that there is a need to increase communication and awareness regarding this gap and to encourage the appropriate use of antibiotics.

### 2.4. Distribution of Antibiotic-Resistant Bacteria in the Dairy Farm Environment and Their Impact on Public Health

**Figure 2:**
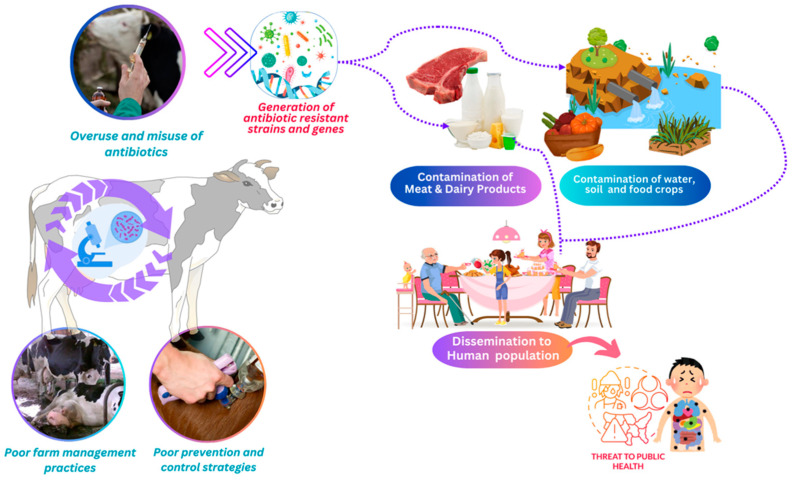
Potential Drivers of Antimicrobial Resistance in Dairy Production Systems.

Dairy farms and other livestock farms are recognized as primary sources of antibiotic-resistant bacteria. These antibiotic-resistant bacteria can survive in the agricultural environment; they infect animals and consequently, milk, meat, and other dairy products, thus posing a potential threat to human beings through foodborne disease. The effects of dairy manure (solid and slurry) on antibiotic-resistant soils have been explored in various studies over the last few years [117,118,119,120,121,122,123,124]. Furthermore, Kim et al. (2021) [125] discussed the emergence of antibiotic-resistant bacteria in the context of livestock production and dairy farms. Thus, it was established that dairy manure contributed to the abundance of ARGs in the soil. This is evidence that ARGs can be released into the environment from dairy farms, thus indicating the importance of observing appropriate waste management measures, as depicted in Figure 2. Other authors have focused on the possibilities of livestock wastewater treatment facilities as sites for the exchange of antibiotic resistance genes between bacteria and the sources of antibiotic-resistant bacteria themselves [126,127,128,129]. This emphasizes the importance of proper waste management practices in dairy farms to control the spread of antibiotic resistance. Measurement and control of AMR in dairy farms are essential for promoting responsible antibiotic use. 

Baker et al. (2022) [120] observed that more research is needed to fight the spread of antimicrobial resistance. They compared the results of primary dairy slurry waste, antimicrobial-resistant bacteria, and genes from both Gram-negative and Gram-positive bacterial pathogens. This also demonstrates that adequate measures should be taken regarding waste disposal because it also contributes to the spread of antimicrobial resistance. The economic loss of AMR in dairy farming includes the costs associated with veterinary service, reduction in milk yield, and mortality. Also, AMR is a big threat to public health since the resistant bacteria can be transmitted from animals to human beings through contact, consuming products from infected animals, especially dairy products, or environmental pathways [120]. This underscores the urgent need for effective strategies to manage AMR in dairy farming.

Some of the measures that are used in the fight against antibiotic resistance in dairy farms include the implementation of antimicrobial stewardship and proper drug use. Veterinary involvement in antimicrobial stewardship training programs is crucial in the dairy industry. These policies on the control of antibiotic resistance should also consider both human and animal uses of antibiotics. Furthermore, exploring alternative treatments for mastitis and other diseases in dairy cows may also help reduce reliance on antibiotics [120].

## 3. Role of Smart Health and PLF for Antibiotic Resistance Mitigation in Dairy Farms

The modern form of livestock farming is precision livestock farming (PLF) which incorporates technological advancements in the feeding system, milking machinery, and management tools to enhance livestock production efficiency. For AMR mitigation, PLF provides the following potential pathways to reduce the use of antimicrobials while keeping the animals’ health and production levels high. For instance, feed intake, behaviour, and other parameters may be automatically tracked and hence early signs of diseases or stress can be treated through diet modification or environmental alteration [130,131,132,133,134,135,136]. Likewise, the robotic milking systems may also notice variations in the composition and quality of the milk produced, which helps in identifying the cases of mastitis and udder infections and thus avoiding the regular use of antibiotics [137,138,139,140]. 

Besides, in dairy farming, digital health monitoring systems may help to identify such vital parameters as body temperature, rumination activity, and activity patterns, to recognize early signs of diseases and prevent their onset [141,142,143]. Hence, through the frequent assessment of the health status of single animals and entire herds, smart health allows for the application of management measures that can help avoid outbreaks of diseases and reduce the use of antimicrobials [142,144,145,146,147]. In addition, such systems collect data that can help in decision-making and the provision of individualized health management for each animal.

PLF systems can then use this information combined with data from sensor networks, electronic health records, and genomic information to get a complete picture of the health of the dairy herds and support management strategies that minimize the use of antimicrobials.

The application of the smart health concept in large-scale dairy farms has also proved to be effective in decreasing the number of antimicrobials used and enhancing animal health and welfare. The role of proper data collection and analysis of antimicrobial drug usage in dairy farms has also been stressed in previous studies [148,149]. To support the process of the antimicrobial administration data, electronic interfaces have been created to perform the calculations of frequencies of the treatments and to assist in tracking and decreasing the use of the antimicrobials [149].

Research has indicated that antimicrobial usage in dairy farms can lead to multidrug resistance in pathogenic bacteria, emphasizing the need for effective antimicrobial stewardship programs [150,151]. Studies have also focused on the relationship between antimicrobial usage and antimicrobial resistance, highlighting the importance of optimizing antimicrobial usage for clinical mastitis and overall udder health improvement [151,152,153].

Attempts have been made to assess and control the use of antimicrobials in dairy cattle farming through surveillance and research to establish effective monitoring systems and guidelines for data collection to minimize the development of resistance [154,155]. Furthermore, studies have also described the trends and intensities of antimicrobial use in dairy herds to assess the present situations and future developments [156].

Moreover, the awareness of animal welfare and certification among consumers and farmers has been examined, along with the importance of welfare assessment and its regularity in monitoring the animal welfare on dairy farms [157,158,159,160]. Some of the monitoring tools that have been used include machine vision-based methods to monitor animal behavior and welfare thus improving the health of dairy cattle [161]. 

However, the use of smart health systems along with the proper collection and analysis of data is vital in large-scale dairy farms in minimizing the use of antimicrobials and improving the welfare of the animals. Information technology, particularly data analytics and AI, are the cornerstones of precision livestock farming. These technologies allow for the gathering of information as well as the merging and analyzing of information from various sources; it can be from the animals themselves, farm management, and even environmental information. By harnessing the power of data analytics and AI, farmers can gain valuable insights into animal health, behavior, and performance, which can inform decision-making and optimize farm management practices. 

Regarding the problem of antibiotic resistance, data analytics, and AI may be highly effective in analyzing patterns and trends concerning antibiotic consumption and resistance on dairy farms. Thus, through reviewing the data on antibiotic use and the treatment results and resistance profiles, the farmers and veterinarians can find out the roles of antibiotic prescriptions in the genesis of resistance and then come up with solutions to prevent the further development of antibiotic resistance [162,163,164]. For instance, data analytics can be used to determine the time or region within the farm where antibiotics are used most often, and thus introduce preventive measures and educate the farmer on the appropriate use of the drugs [156,163,165,166]. Besides, AI predictive models can help in the screening of disease outbreaks and identifying animals that are more prone to contracting antibiotic-resistant infections [167,168,169]. Applying these models, the information about animal health, the environment, and the previous history of diseases is used to predict the possible risks and prevent diseases without using antibiotics. Thus, it is possible to prevent the occurrence and further dissemination of antibiotic-resistant bacteria in dairy farms with this approach to disease control.

## 4. Challenges and Opportunities in the Adoption of the Smart Health Concept and PLF in Dairy Farms

Although digital health monitoring and precision livestock farming (PLF) offer several advantages, their broad adoption in dairy farming is hindered by various obstacles, such as economic constraints, technological restrictions, and farmers’ reluctance to embrace change. Implementing standard regulations in the dairy farming business is challenging due to the wide range of methods and industry sizes across various nations [113]. The substantial upfront expenses associated with adopting PLF technology may re-strict small-scale farmers’ ability to use these advancements [170,171,172,173]. For this reason, the supply of funds and incentives by governments and agricultural organizations is crucial for the enhancement of Precision Livestock Farming (PLF). For this reason, the supply of funds and incentives by governments and agricultural organizations is crucial for enhancing Precision Livestock Farming (PLF). However, there are concerns on data privacy, interoperability, and standardization which are other challenges that affect the use of these technologies on a large scale. Nevertheless, as sensors, data analytics, and connectivity systems evolve, the challenges that hinder the implementation of these technologies are being progressively tackled. This is creating new possibilities for innovation and cooperation within the dairy sector.

By promoting collaborations among academics, industry stakeholders, and policymakers, focused endeavours may be undertaken to expedite the adoption of smart health and precision livestock farming techniques and successfully reduce antimicrobial resistance in dairy farms.

## 5. Conclusions

To sum up, both concepts of smart health and precision animal farming hold great promise in improving dairy farming operations and solving the contemporary issue of antibiotic resistance. Using information and tools can help dairy farmers reduce the occurrence of diseases in animals and subsequently the use of antibiotics. However, to effectively harness these technologies all the stakeholders have to also work towards solving the issues regarding the technology, economics, and the legal framework. The policymakers can promote sustainable agricultural practices and ensure fair investments in the digital platform and innovation. Nevertheless, there is still a need for additional initiatives to progress the current level of expertise and use of Smart Health and Precision Livestock Farming (PLF) technology in the context of dairy farming. Therefore, by using technical breakthroughs and sustainable management approaches, the dairy industry has the potential to lead the way in combating antimicrobial resistance and ensuring the production of safe and nutritious food products for society.

## Data Availability

Not applicable.
